# Development and validation of an endoscopic diagnostic model for sessile serrated lesions based on machine learning algorithms

**DOI:** 10.3389/fmed.2025.1665079

**Published:** 2025-10-15

**Authors:** Xinying Yu, Lianyu Li, Qiang He

**Affiliations:** ^1^Department of Gastroenterology, Beijing Tiantan Hospital, Capital Medical University, Beijing, China; ^2^Huazhong University of Science and Technology, Wuhan, China

**Keywords:** sessile serrated lesion, artificial intelligence, machine learning, colorectal polyps, hyperplastic polyps

## Abstract

**Background and aims:**

Sessile serrated lesions (SSLs) are morphologically subtle and often misclassified as hyperplastic polyps (HPs), increasing colorectal cancer risks. We developed a machine learning (ML) model to improve endoscopic SSL diagnosis.

**Methods:**

Three hundred and eighty-six colorectal polyps (135 SSLs, 251 HPs) with histologically confirmed were retrospective analyzed and divided into a training set and a test set. Multiple ML classification models were applied for a comprehensive analysis. SHapley Additive exPlanations (SHAP) for model contribution were plotted, and the model results were interpreted by calculating the contribution of each feature to the prediction results.

**Results:**

Comparative analysis revealed that the shrinkage method based on penalisation and post-estimation model fit (R^2^ Shrinkage) model demonstrated superior performance in the SSL diagnostic task, with an average accuracy of 84.7% ± 7.7, a specificity of 71.2% ± 15.0, a sensitivity of 92.7% ± 4.1 and *F*_1_-score of 88.5% ± 6.2. The results revealed that the area under the curve (AUC) values based on both the validation and test sets eventually stabilized at approximately 0.90, indicating the reliable predictive performance of the model. By constructing individualized SHAP plots, we established quantitative diagnostic criteria: when the lesion size was >8 mm, there was a mucus cap, the lesion was located in the right half of the colon, SSL was predicted with a probability of more than 85%; otherwise, HP tended to be diagnosed.

**Conclusion:**

This study represents the first application of an ML algorithm techniques to the endoscopic classification of serrated polyps. The lesion size, mucus cap and lesion location are key features for the endoscopic diagnosis of SSL.

## 1 Introduction

Colorectal serrated lesions are a type of neoplastic lesion with significant morphological heterogeneity and molecular biological characteristics. These lesions include sessile serrated lesions (SSLs), hyperplastic polyps (HPs), and traditional serrated adenomas (TSAs) (1). In recent years, with in-depth studies of the pathogenesis of colorectal cancer (CRC), the clinical importance of SSL as a key prodromal lesion of the “serrated neoplasia pathway” (2, 3) has become increasingly prominent. Studies have shown that approximately 20% of sporadic colorectal cancers originate from SSLs, and these lesions have the potential to progress rapidly to highly dysplastic or even invasive cancer (4, 5). Therefore, early identification of SSLs and complete resection are crucial for reducing the incidence of CRC. HPs, on the other hand, are nonneoplastic polyps that usually carry no potential for malignancy and generally do not require intervention. However, HPs are very similar to SSLs under endoscopy. The typical features of SSLs, such as mucus caps, unclear boundaries, and cloud-like surfaces, are more easily distinguishable from those of TSAs but often overlap with those of HPs (6, 7), especially in conventional white light endoscopy (WLE) examinations, which lack support from narrowband imaging (NBI), optically enhanced endoscopy, or magnifying endoscopy. The risk of misdiagnosis or missed diagnosis is relatively high (8). Although several studies have proposed endoscopic diagnostic criteria for SSLs, such as the Japan NBI Expert Team (JNET) classification (9), these criteria rely on advanced imaging techniques and are difficult to widely promote in primary care institutions. In addition, the feature combinations of existing diagnostic systems are complex, and clinicians still exhibit a high degree of subjective judgment bias in practice. Therefore, developing a diagnostic system based on the characteristics of conventional WLE to improve the detection rate and diagnostic accuracy of SSLs is highly valuable for optimizing clinical management strategies.

The rapid development of computer-aided diagnostic (CAD) systems based on machine learning (ML) techniques provides new ideas for addressing this challenge. As an important branch of artificial intelligence, ML (10) offers a powerful set of algorithms for learning, adapting to, predicting and analyzing massive amounts of medical data (11, 12) to provide strong support for clinical decision-making. These methods perform medical diagnostic tasks by using feature extraction techniques, such as logistic regression analysis (13), for feature screening and by using classifiers for prediction and classification. CAD systems based on ML techniques provide strong technical support for medical diagnosis through efficient feature extraction and advanced classification algorithms. In this study, through retrospective cohort analysis, independent predictors of SSL endoscopy diagnosis were screened, an endoscopy diagnostic model that does not rely on advanced imaging techniques using ML methods was constructed, and efficient SSL recognition tools were provided. Therefore, the main contributions of this paper are summarized as follows: (1) We present the first use of a machine approach to classify serrated polyps in endoscopic images. This approach led to significantly increased classification accuracy; (2) A systematic comparison of extreme gradient boosting (XGBoost) (14), logistic regression analysis (Logistic), least absolute shrinkage and selection operator (LASSO) (15), Shrinkage method based on penalisation and post-estimation model fit (R^2^ Shrinkage) (16), light gradient boosting machine (LightGBM) (17), random forest (18), adaptive boosting (AdaBoost) (19), multilayer perceptron (MLP) (20), support vector machine (SVM) (21), K-nearest neighbor (KNN) (22), and Gaussian naive Bayes (GNB) (23) was performed. The results showed that although some models perform similarly in specific metrics, R^2^ Shrinkage shows a clear advantage in overall diagnostic performance; and (3) the SHapley additive exPlanations (SHAP) (24) method was applied to conduct a comprehensive interpretability analysis of the ML models and quantify the contribution of each feature to the model’s decision-making by calculating the SHAP values.

## 2 Methods

### 2.1 Case data

Clinical data from patients who underwent colonoscopy and endoscopic colonic polyposectomy at Beijing Tiantan Hospital, Capital Medical University, from January 2021 to December 2024 were collected and retrospectively analyzed. All patients included in the study were consecutively enrolled. The inclusion criteria were as follows: (1) patients who had undergone total colonic examination; colonic polyps were identified, and polyposectomy was performed, including endoscopic mucosal resection (EMR) or endoscopic submucosal dissection (ESD); and (2) patients whose postoperative pathology confirmed serrated lesions or hyperplastic polyps. The exclusion criteria were as follows: (1) postoperative pathological diagnosis of adenoma, cancer, normal mucosa, or other nonserrated lesions; (2) poor intestinal preparation that affects observation (Boston score less than 6); and (3) missing clinical or pathological data.

### 2.2 Endoscopic procedure

Colonoscopy and treatment for all patients were performed by expert endoscopists with at least 5 years of experience in endoscopic treatment. All patients were given a standardized bowel preparation protocol. The specific medication was 4 boxes of compound polyethylene glycol electrolyte powder (6 bags per box, each bag containing 13.125 g of polyethylene glycol 4000). The patients were required to take the powder dissolved in 3,000 mL of warm water at a uniform speed 4–6 h before the endoscopy. After completing the intestinal preparation, the patients underwent colonoscopy under intravenous anesthesia. During the colonoscopy, all polyps identified by white light imaging were rinsed with water, and photos before and after rinsing were taken and observed and evaluated using optical enhancement (OE). Subsequently, endoscopic treatment methods, mainly EMR and ESD, were selected on the basis of the experience of the endoscopist, and specimens were collected for pathological evaluation after the operation.

### 2.3 Definitions and collection of observation indicators

The location, size, shape and endoscopic diagnosis results of each lesion that was successfully evaluated and resected were recorded. Lesion location was categorized as either the proximal colon or the distal colon. The proximal colon includes the ileocecal region, ascending colon, and transverse colon. The distal colon includes the descending colon, sigmoid colon and rectum. Lesion size was estimated by comparison with the snare opening. Lesion morphology was endoscopically classified according to the Paris classification criteria, including pedicled type (0-Ip), subpedicled type (0-Is), superficial protuberant type (0-IIa), superficial flat type (0-IIb), superficial depressed type (0-IIc), depressed type (0-III), and superficial protuberant + superficial depressed type (0-IIa + IIc). Under white light and OE, characteristics such as mucus caps, a cloud-like or red surface, unclear boundaries, surface microvessel thickening, and crypt opening dilation were assessed. A mucus cap is defined as a large amount of mucus or feces covering the surface of a lesion. Cloud-like surfaces are characterized by granular or nodular protrusions resembling cumulus clouds. Red surfaces were identified when lesion coloration turned red when observed under white light. Indistinct boundaries are defined as lesion boundaries being blurred and lacking a clear demarcation. Surface microvessel thickening is defined as the presence of tortuous and thickened microvessels under OE. Crypt opening dilation is defined as nonuniform expansion of the crypt morphology under OE. The characteristics of the polyps are shown in Table 1.

**Table 1:** Description of the characteristics of the colonic polyps studied.

Feature number	Feature name	Feature description
1	Part	1-Right half colon; 2-Left colon
2	Size	Units mm
3	Endoscopic typing	1-Ip, 2-Is, 3-IIa, 4-IIb, 5-IIc
4	Slime cap	0-None; 1-Yes
5	Thickening of the surface vessels	0-None; 1-Yes
6	Red surface	0-None 1-Yes
7	Blurred boundaries	0-None; 1-Yes
8	Enlarged crypt openings	0-None; 1-Yes

### 2.4 Construction and evaluation of predictive models

After the features were selected from all the independent variables, the enrolled patients were divided into a training set and a test set. Multiple ML classification models were applied for a comprehensive analysis to compare the importance of each metric in the training and test sets. The results were evaluated and validated using the best model. The steps are as follows: (1) Feature factor screening: First, feature selection was conducted through least absolute shrinkage and selection operator (LASSO) regression combined with multivariate logistic regression analysis, and feature factors with statistical significance (*p* < 0.05) were retained as predictive variables for subsequent modeling. (2) Data division: SSL patients were randomly divided into a training set and a test set at a 7:3 ratio, with 270 cases in the training set and 116 cases in the test set. (3) Classification multimodel synthesis analysis: XGBoost, Logistic, LASSO, R^2^ Shrinkage, LightGBM, RF, AdaBoost, MLP, SVM, KNN and GNB methods were constructed. By evaluating key metrics such as accuracy, specificity, sensitivity, and *F*_1_-score for each model based on the training and test sets, the model’s discriminative ability was comprehensively evaluated in combination with the area under the receiver operating characteristic curve (AUC-ROC), and the optimal prediction model was finally selected (25). (4) Training, validation and testing of the optimal model: We performed 10 cross-validation using the training set and evaluate it with the test set. ROC learning curves and confusion matrices were plotted to evaluate model fit and stability for both the training and validation sets. (5) Model interpretability using SHAP: SHAP interpretations were plotted for model importance and contribution, and the model results were interpreted by calculating the contribution of each feature to the prediction results (26).

## 3 Results

### 3.1 Patient information

**Figure 1:**
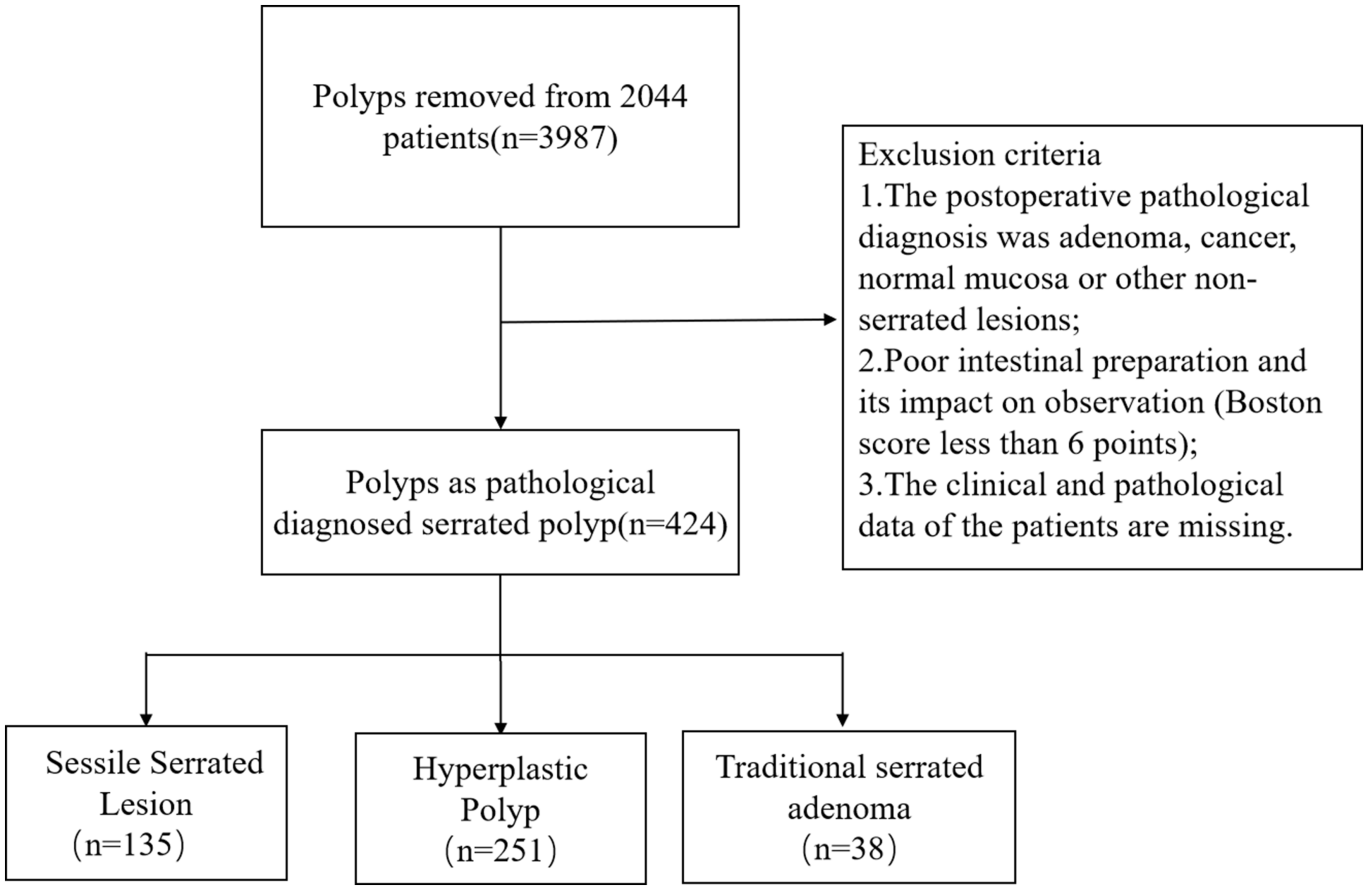
Flowchart of the study.

A total of 2,044 patients who underwent colonoscopy and endoscopic polyposectomy were included in the study. Among the 3,987 polyps removed from these patients, a total of 424 polyps were pathologically diagnosed as serrated polyps and were classified as SSLs, HPs, or TSAs according to the World Health Organization (WHO) classification criteria (1). A total of 135 cases of SSL and 251 cases of HP were included in this study. Figure 1 shows the flowchart of the study. This study did not include 38 cases of TSA. The reason is that TSA usually has typical endoscopic morphological features, such as villous or papillary protrusions, and the difficulty of clinical differentiation is relatively low. To focus on the core issue of SSL and HP, which is highly difficult to distinguish and has significant clinical demands, the model construction of this study is only for SSL and HP lesions, in order to enhance the performance and practicality of the model in key differentiation tasks.

### 3.2 Screening of SSL diagnostic characteristic factors

In this study, LASSO regression analysis (with the SSL category as the dependent variable) was used for feature selection of the independent variables, a method that effectively prevents overfitting by compressing the variable coefficients to solve multicollinearity problems (27). On the basis of the LASSO screening results, the associations between each clinical feature and the target outcome were evaluated through multivariate logistic regression analysis. The final model included variables such as location, size, endoscopic classification, mucus cap, surface vascular thickening, red surface, boundary blurring, and enlarged crypt openings. The analysis results (shown in Table 2) revealed that lesion location (coeff. = 0.8602, *p* < 0.001) and surface vessel thickening (coeff. = 0.8589, *p* = 0.011) were significantly positively correlated with the target outcome, whereas lesion size (coeff. = −1.3989, *p* < 0.001) and mucus cap (coeff. = −0.7809, *p* = 0.003) were significantly negatively correlated. Endoscopic classification (coeff. = 0.3867, *p* = 0.049) was statistically significant, but the effect size was relatively small. Notably, red surfaces (*p* = 0.096) and blurred boundaries (*p* = 0.051) did not reach the traditional significance threshold, but their clinical significance is still worthy of attention. No significant differences were observed in the number of enlarged crypt openings (*p* = 0.375). These findings provide an important basis for endoscopic clinical decision-making, and a focus on characteristics such as lesion location, size, mucus cap, and thickening of surface vessels during the assessment process is recommended.

**Table 2:** Logistic regression analysis (multivariate analysis).

Variable	Coeff.	Std. err.	*z*	*p* > |*z*|	95% CI lower	95% CI upper
Part	0.8602	0.184	4.664	<0.001	0.499	1.222
Size	−1.3989	0.418	−3.348	<0.001	−2.218	−0.580
Endoscopic typing	0.3867	0.196	1.970	0.049	0.002	0.771
Mucus cap	−0.7809	0.263	−2.972	0.003	−1.296	−0.266
The surface vessels thicken	0.8589	0.338	2.543	0.011	0.197	1.521
Red surface	−0.6028	0.362	−1.667	0.096	−1.312	0.106
Blurred boundaries	−0.8159	0.418	−1.952	0.051	−1.635	0.003
Enlarged crypt openings	0.3501	0.394	0.888	0.375	−0.423	1.123

### 3.3 Classification multimodel synthesis analysis

Table 3 summarizes the performance metrics of various machine learning algorithms in SSL diagnostic tasks, including accuracy, specificity, sensitivity, and *F*_1_-score. While the overall accuracy of most algorithms is similar, there are notable differences in sensitivity and specificity. Specifically, R^2^ Shrinkage achieves the best balance between sensitivity (92.7% ± 4.1) and specificity (71.2% ± 15.0), outperforming other algorithms. Logistic regression, though exhibiting the highest sensitivity (92.3% ± 4.3), shows lower specificity, whereas XGBoost and LightGBM offer slightly better specificity but at the cost of reduced sensitivity. These differences highlight the trade-off between false positives and false negatives in SSL diagnosis. The advantage of R^2^ Shrinkage lies in its adjustment mechanism based on penalization and post-estimation model fit (R^2^-based shrinkage), which reduces overfitting while improving the model’s ability to correctly identify minority positive samples, thus enhancing the balance between sensitivity and specificity. As a result, R^2^ Shrinkage achieves the highest *F*_1_-score (88.5% ± 6.2), demonstrating its superior overall diagnostic performance.

**Table 3:** Performance evaluation of the XGBoost, Logistic, LASSO, R^2^ Shrinkage, LightGBM, RF, AdaBoost, SVM, KNN, and GNB algorithms in SSL diagnosis.

Algorithm	Accuracy	Specificity	Sensitivity	*F*_1_-score
XGBoost	84.7 ± 6.6	76.6 ± 13.8	89.5 ± 5.3	88.4 ± 5.6
Logistic	84.2 ± 7.3	70.0 ± 14.1	92.3 ± 4.3	88.2 ± 6.0
LASSO	84.2 ± 7.3	70.0 ± 14.1	92.3 ± 4.3	88.2 ± 6.0
R^2^ Shrinkage	**84.7 ± 7.7**	**71.2 ± 15.0**	**92.7 ± 4.1**	**88.5 ± 6.2**
LightGBM	83.4 ± 6.7	76.0 ± 13.8	88.0 ± 6.3	87.1 ± 5.6
RF	83.4 ± 7.0	73.9 ± 13.5	89.1 ± 5.2	87.2 ± 5.9
AdaBoost	84.7 ± 6.4	75.4 ± 13.2	90.3 ± 5.3	88.3 ± 5.3
MLP	83.7 ± 8.4	73.5 ± 15.9	90.0 ± 5.4	87.6 ± 6.8
SVM	84.0 ± 5.6	73.1 ± 12.3	89.9 ± 5.4	87.7 ± 4.9
KNN	83.9 ± 5.7	77.5 ± 13.0	87.6 ± 4.1	87.5 ± 4.9
GNB	82.2 ± 7.5	67.8 ± 10.7	90.3 ± 4.9	86.5 ± 6.4

The bold values represented that R2 Shrinkage model demonstrated greater performance and reliability.

**Figure 2:**
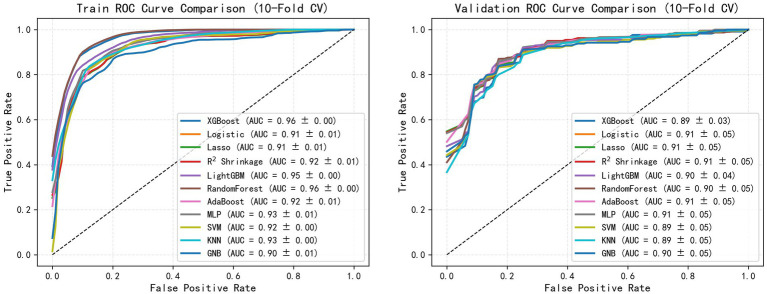
Comparative analysis of the ML models.

To quantitatively evaluate the classification performance of each model, the ROC curve of the above algorithm in the colonic polyp identification task was plotted, and the AUC value was calculated. As shown in Figure 2, all models achieved high classification performance, with AUC values concentrated between 0.89 and 0.91. On the training set (Figure 2A), ensemble methods such as XGBoost and Random Forest reached the highest mean AUC (0.90–0.91), indicating strong fitting ability. However, on the validation set (Figure 2B), penalized regression models (Logistic, LASSO, R^2^ Shrinkage) and MLP maintained relatively higher and more consistent AUCs (0.91), whereas tree-based models showed a slight decline (0.89–0.90). These results suggest that, while most algorithms performed similarly, R^2^ Shrinkage in particular achieved a favorable balance between accuracy and generalizability, highlighting its potential superiority in SSL diagnostic applications.

### 3.4 Best model construction and evaluation

**Figure 3:**
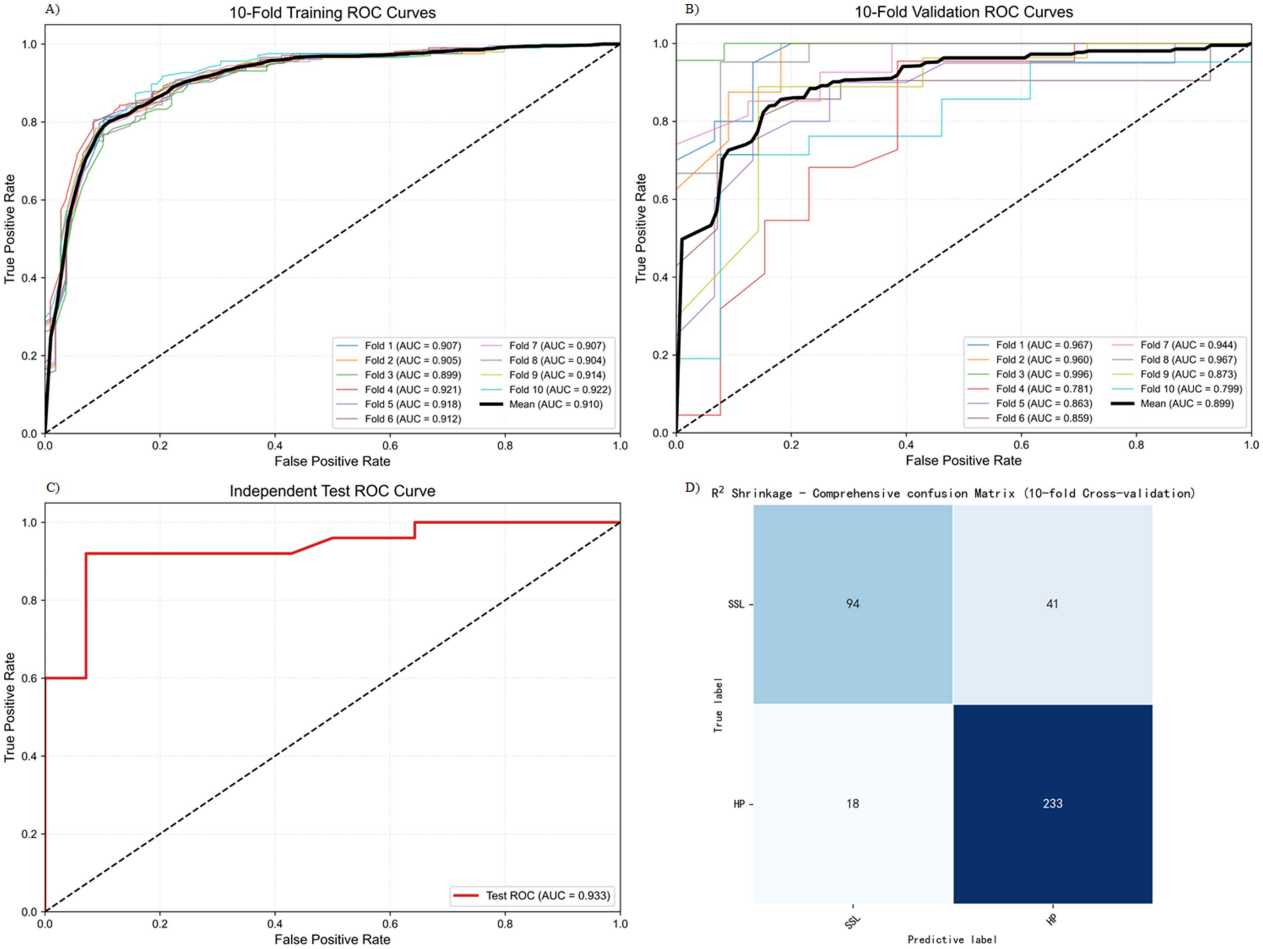
Training and testing results of the R2 Shrinkage model. **(A)** 10-fold training ROC curve. **(B)** 10-fold validation ROC curve. **(C)** Independent test ROC curve. **(D)** The cumulative confusion matrix.

We conducted 10-fold cross-validation on the training set using the R^2^ Shrinkage algorithm, and the results revealed that the average AUC of the training set reached 0.910 (ranging from 0.899–0.922), the average AUC of the validation set was 0.899 (ranging from 0.781–0.996), and the AUC of the test set was 0.933 (Figures 3A–C). As illustrated in Figure 3, the R^2^ Shrinkage model exhibited strong and consistent discriminative performance across all datasets, with no signs of overfitting. The cumulative confusion matrix (Figure 3D) further confirmed its robust diagnostic capability, correctly classifying 94 SSL cases and 233 HP cases. These results demonstrate that the R^2^ Shrinkage model generalizes well and shows practical utility for SSL identification.

### 3.5 SHAP for model interpretability

**Figure 4:**
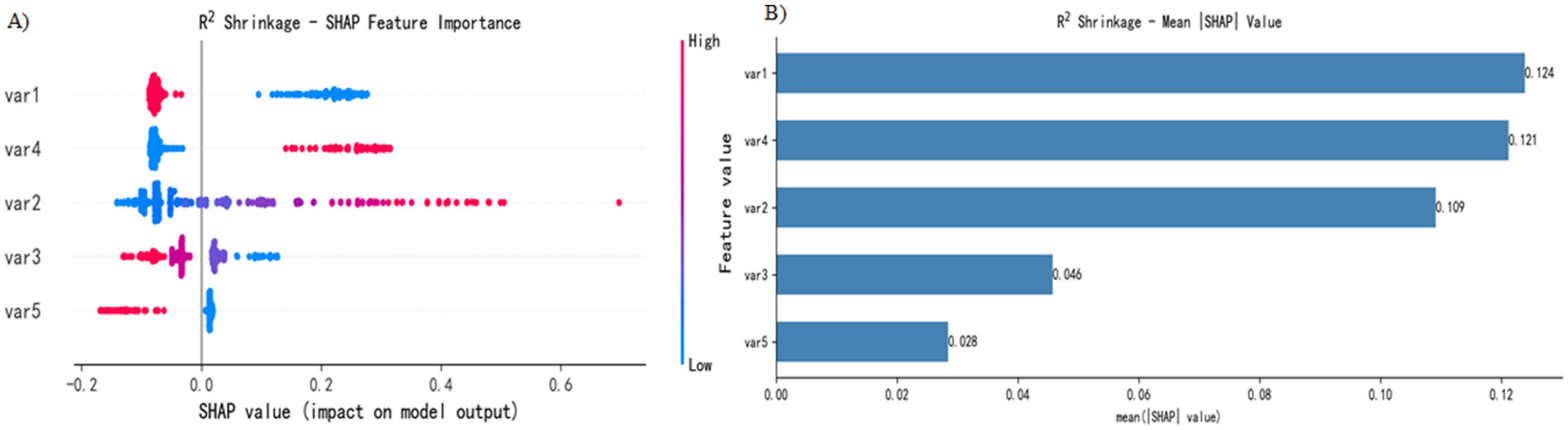
SHAP interpretation. **(A)** Attributes of features in the SHAP analysis. Each point represents a feature, and the SHAP values are plotted on the *x*-axis. The red dots represent high eigenvalues, and the blue dots represent low eigenvalues. **(B)** Feature importance sorted by SHAP value; the matrix diagram describes the importance of each covariate in the final prediagnosis process.

The SHAP analysis conducted in this study revealed patterns in the contribution of key features to the model’s outputs (Figure 4). The visualization results highlight the relative importance of five key features and their predicted trends: the *x*-axis represents the SHAP value (a positive value indicates a positive correlation, and a negative value indicates a negative correlation), and the color gradient represents the magnitude of the feature value (red, high value; blue, low value). This two-dimensional visualization clearly shows how feature contribute to the model predictions.

Figure 4A shows the five most important features in the model. Features 1 (location), 4 (mucus cap), 2 (size), 3 (endoscopic typing), and 5 (surface vascular thickening) were identified as key predictors in SSL diagnosis. Specifically, (1) the high values of features 2 and 4 are distributed mainly in positive regions, indicating that a larger lesion size and the presence of a mucus cap increase the probability of SSL diagnosis; (2) the high values of features 1, 3 and 5 are concentrated in negative areas, suggesting that lesions located in the left half of the colon, specific endoscopic subtypes and the presence of vascular thickening reduce the probability of SSL diagnosis; and (3) feature 3 has the widest distribution of SHAP values, indicating that its influence is more complex.

Figure 4B presents the analysis results of feature importance on the basis of the absolute average SHAP value. In this study, the R^2^ Shrinkage algorithm was used to construct a classification model, and the SHAP method was employed to calculate the contribution of each feature to the model predictions. By calculating the global mean absolute SHAP value, we obtained the feature importance ranking: feature 1 (0.124) was the most influential, followed by feature 4 (0.121), feature 2 (0.109), feature 3 (0.046), and feature 5 (0.028), which had the lowest contribution. This analysis not only objectively identified the key predictive features but also significantly enhanced the interpretability of the model, providing an important reference for subsequent research and clinical application.

## 4 Discussion

In recent years, advances in molecular biology research has highlighted the clinical importance of SSLs as core precursor lesions of the “serrated carcinogenesis pathway” in colorectal cancer. However, the morphological manifestations of SSLs are morphologically subtle, and some cases are missed because of the lack of typical bulges or color changes. Therefore, some studies suggest that CRC that occurs after colonoscopy screening may develop from missed and untreated serrated lesions (28), and colonoscopy is not as effective in screening the right half of the colon as it is for the left half (29). Several studies have confirmed that interphase CRC often occurs in the proximal colon and is associated with the serrated carcinogenesis pathway (30). Therefore, the current view holds that serrated lesions play an important role in the development of CRC. Given the high carcinogenic potential of SSLs, clinical guidelines emphasize complete resection of right hemicolonic serrated lesions ≥5 mm and shortening the follow-up period to 3 years (31, 32) to prevent their progression to invasive cancer. In this study, the detection rate of SSL was 3.3% (135/3,987), which was relatively low compared with previous studies. It mainly reflects the historical limitations of insufficient understanding of SSL and the lack of unified diagnostic criteria in the early stage of the included study. With the popularization of pathological diagnosis norms and the strengthening of training in recent years, the diagnostic accuracy of SSL has significantly improved.

Previous studies have indicated that SSLs are located mostly in the right colon, their diameters are often greater than 5 mm, and endoscopically, they often grow in a flat or broad-based manner and are easily confused with the microvesicular or goblet cell subtypes of HPs (33). As a result, these lesions are prone to be overlooked during endoscopy, leading to a missed diagnosis. Therefore, accurate identification of SSLs under endoscopy is a key step in reducing the incidence of colorectal cancer. Hazewinkel et al. (8) summarized the endoscopic characteristics of SSLs as unclear boundaries, cloud-like surfaces, black spots in the crypts under NBI, irregular shapes, pit pattern II-O type gladular duct openings and normal vascular density. Unclear boundaries and white cumulus surfaces, black spots and irregular shapes within the crypts were identified as independent predictors during NBI examination. However, the diagnostic efficacy of a single feature is limited, and assistive techniques such as magnifying endoscopy or staining endoscopy are not yet widespread, resulting in a higher rate of missed diagnoses in clinical practice. Although several studies have attempted to increase diagnostic accuracy through multiparameter models, most of these models rely on high-resolution or magnifying imaging techniques (34) and are difficult to adopt in primary care. Traditional research methods and statistical methods encounter certain limitations in dissecting these complex factors. This study aims to build an endoscopic classification prediction model of SSLs through ML techniques to address the high rate of missed diagnoses and the difficulty in differentiating SSLs from HPs due to their subtle morphology, improve the early accurate recognition rate, provide technical support for blocking the serrated sawtooth carcinogenesis pathway and optimizing clinical treatment and follow-up strategies.

This study introduced an SSL intelligent diagnostic model based on ML algorithms, with a focus on differentiating between SSL and HP diagnoses. A systematic comparison of the diagnostic performance of nine algorithms, including XGBoost, Logistic, LASSO, R^2^ Shrinkage, LightGBM, random forest, AdaBoost, MLP, SVM, KNN and GNB, revealed that the R^2^ Shrinkage model performed the best, with a diagnostic accuracy rate of 84.7% ± 7.7. The specificity was 71.2% ± 15.0, the sensitivity was 92.7% ± 4.1 and the *F*_1_-score was 88.5% ± 6.2. As the first study to apply machine learning techniques to endoscopic classification, this method enables precise differentiation between SSLs and HPs, providing new ideas for clinical diagnosis. Although differences exist in datasets and evaluation protocols across studies, the performance of our model can be contextualized with previous machine learning and deep learning studies on SSL diagnosis (see Supplementary Tables 1, 2), demonstrating comparable or superior potential (35–39). The excellent performance of R^2^ Shrinkage is attributed mainly to the following: (1) the R^2^ Shrinkage method can effectively adjust model coefficients to account for potential overfitting; and (2) this shrinkage improves the stability and generalizability of the diagnostic model across different datasets. This study provides a reliable intelligent diagnostic approach for the classification of serrated polyps.

In terms of model interpretability studies, SHAP analysis was applied to systematically evaluate the contribution of each clinical feature to SSL diagnosis. By constructing a global bee colony map and an average absolute SHAP plot, we visually demonstrated the importance ranking of different features in model decision-making and the direction of their influence. The results revealed the following: (1) Lesion size (absolute mean SHAP = 0.109) and the presence of a mucus cap (0.121) were significantly positively correlated with SSL classification. When a diameter >8 mm or mucus cap was present, the SHAP value was positively offset, suggesting an increased probability of SSL diagnosis. (2) The left hemicolonic region (0.124), specific endoscopic classification (0.046), and surface vascular thickening (0.028) were negatively correlated with SSLs, among which the endoscopic classification had the greatest dispersion of the SHAP value distribution, reflecting its nonlinear effect characteristics. (3) Surface vascular thickening contributed the least (0.028). By constructing individualized SHAP maps, we established quantitative diagnostic criteria and proposed the three most contributing indicators as the basis for SSL diagnosis: when the lesion size was >8 mm, there was a mucus cap, and when the lesion was located in the right half of the colon, the probability of an SSL diagnosis was more than 85%; otherwise, the model predicts HP. The model innovatively reveals the interaction patterns among features, its diagnostic efficacy is highly consistent with clinical guidelines, and its feature of not relying on magnifying endoscopy is particularly suitable for primary care institutions and routine endoscopic examination scenarios, providing efficient and objective decision support for SSL differentiation. In addition, the model constructed in this study provides a quantitative basis for the clinical diagnosis of SSL by integrating key features such as the location, size and mucus cap of the lesion. This tool helps to reduce the missed diagnosis rate of SSL, improve the diagnostic consistency among different operators, and especially provides a reference for the real-time decision-making of endoscopists.

There are certain limitations in this study. First, in terms of the study design, a retrospective study method was adopted. Retrospective data lack the consistency to standardize endoscopic procedures such as image quality and shooting angle, and individual differences among patients, such as intestinal preparation quality and combined medication were not effectively corrected, which might amplify the differential error between SSLs and HPs. Second, there was an imbalance in the sample size. The sample size of the HP group was greater than that of the SSL group, which led to the ML model overfitting to most classes (HP) and reducing its classification sensitivity to SSLs. In addition, the model was only validated using retrospective datasets, and its actual clinical efficacy was not tested in multicenter, prospective cohorts, which may overestimate the classification accuracy. Prospective multicenter studies are needed in the future to expand sample diversity and use oversampling synthetic minority oversampling technique (SMOTE) or generative adversarial networks (GANs) to address the imbalance between classes. In addition, manual extraction of endoscopic features may introduce subjectivity and individual differences due to the varying experiences and judgment criteria of observers. In future research, we plan to expand the scale of the dataset and consider exploring the end-to-end application of deep learning for adaptive feature extraction to further enhance the objectivity and generalization ability of the model.

## 5 Conclusion

This study is the first to apply ML algorithms to the endoscopic classification of serrated polyps and can differentiate SSLs in non-magnifying endoscopic clinical scenarios, supporting diagnosis and providing a feasible and efficient new method for this purpose. Compared with those of the other studied models, the R^2^ Shrinkage model demonstrated greater performance and reliability, yielding higher accuracy, specificity, sensitivity, and *F*_1_-score. In addition, the SHAP value analysis revealed that a lesion size >8 mm, the presence of a mucus cap, and the lesion location in the right half of the colon were key features for the endoscopic identification of SSLs.

## Data Availability

The original contributions presented in the study are included in the article/Supplementary material, further inquiries can be directed to the corresponding author.
