# Supplementary material for: Development and validation of an endoscopic diagnostic model for sessile serrated lesions based on machine learning algorithms
Source: Front Med (Lausanne). 2025 Oct 15;12:1665079. doi: 10.3389/fmed.2025.1665079 (PMC12568591; doi:10.3389/fmed.2025.1665079)
Supplement: Supplementary file 1 [file Data_Sheet_1.docx]

**Supplementary information**

**Development and validation of an endoscopic diagnostic model for sessile serrated lesions based on machine learning algorithms**

Qiang He et al.

1. The SSL vs HP using deep learning and machine learning for generalized diagnostics

This appendix compares existing studies on the diagnosis of sessile serrated lesions (SSLs) using traditional machine learning and deep learning methods. Supplementary Table 1 summarizes the methodological characteristics of the study, including the author, year, data source, input type, sample size, annotation standard and method, etc. Supplementary Table 2 presents the performance indicators (accuracy, sensitivity, specificity and AUC, etc.) of each study. It should be noted that the differences among various studies in terms of data sources, annotation standards and evaluation processes limit direct numerical comparisons. However, these tables can still provide a horizontal descriptive assessment, enabling readers to understand the performance trends and methodological diversity in SSL diagnostic research.

**Supplementary Table 1** ethodological and dataset characteristics of traditional machine learning and deep learning research

| Author | Year | Data source | Input type | Sample size | Marking standard | Method |
| --- | --- | --- | --- | --- | --- | --- |
| Houwen et al.[35] | 2023 | Eight Dutch hospitals and one Spanish hospital | NBI static image | Training: 2,637 pieces;  Verification: 423 polyps (300 adenomas, 41 SSL, 82 HP) | The gold standard of  pathology | POLAR system: YOLOv4 positioning + SIFT feature classification + real-time CADx |
| Zhou et al.[36] | 2020 | Sichuan Provincial People's Hospital | Image + video | LST: 184 patients, 1,451 images; Among them, 184 cases were SSA/P | Pathology Gold Standard + Expert Consensus | EndoScreener (CNN-based CADe) + sensitivity analysis per frame/per lesion |
| Soo & Koh [37] | 2023 | Shengkang General Hospital, Singapore | Real-time colonoscopy video | The total number is not specified (clinical prospective study). | Pathological Gold Standard + JNET classification | GI Genius™ (Medtronic) Real-time AI detection System |
| Kato et al. [38] | 2024 | Showa University North Yokohama Hospital, Japan | NBI static image | There were 500 polyps, including 346 adenomas/adenocarcinomas, 38 SSLS, and 116 non-neoplastic polyps | Pathological Gold Standard + JNET classification | A before-and-after comparative study of EndoBRAIN-X (Cybernet) CADx system and multiple film readers |
| Yoon et al. [39] | 2022 | Gangnam Medical Center, Seoul National University Hospital | White light endoscopy image | The training set contains 4,397 polyp images, including 2,528 adenomas, 222 SSLS, 1,585 HPS, and 88 others | The gold standard of pathology | StyleGAN2 generates SSL images + YOLOv3 detection model + data augmentation strategy |

**Supplementary Table 2** Performance indicators of traditional machine learning and deep learning research

| Author | Accuracy | sensitivity | specificity | AUC | threshold | method |
| --- | --- | --- | --- | --- | --- | --- |
| Houwen et al.[35] | 79.4% (POLAR) | 89.4% (POLAR) | 37.8% (POLAR) | - | It was comparable to the performance of endoscopists (with an accuracy rate of 83.0%), but did not meet the PIVI/SODA standards | POLAR system: YOLOv4 positioning + SIFT feature classification + real-time CADx |
| Zhou et al.[36] | - | -94.07% (each figure, LSTs) | - | - | The sensitivity of SSL per frame is 84.10%, and the sensitivity per lesion is 100% | EndoScreener (CNN-based CADe) + sensitivity analysis per frame/per lesion |
| Soo & Koh [37] | - | - | - | - | The case report does not provide numerical indicators and emphasizes the practicality of AI-assisted SSL detection | GI Genius™ (Medtronic) Real-time AI detection System |
| Kato et al. [38] | 91.8%  (CADx assistance) | 95.6% (CADx assistance) | 78.9% (CADx assistance) | - | CADx significantly enhances the diagnostic confidence and high-confidence diagnosis rate of endoscopists | A before-and-after comparative study of EndoBRAIN-X (Cybernet) CADx system and multiple film readers |
| Yoon et al. [39] | 93.55% (GAN-aug2) | 95.44% (GAN-aug2) | 90.10% (GAN-aug2) | 0.96 | After GAN enhancement, the SSL detection sensitivity increased by 17.5%, and the AP rose to 0.9302 | StyleGAN2 generates SSL images + YOLOv3 detection model + data augmentation strategy |
